# Unplanned Clinic Attendance, Readmission, and Reoperation in the First 12 Months Postoperatively Following Hip Hemiarthroplasty for Acute Hip Fractures: Who Is At Risk?

**DOI:** 10.7759/cureus.6128

**Published:** 2019-11-11

**Authors:** Rafia Ghani, Muhammad Usman, Omer Salar, Abdul M Khan, Jamila Karim, Edward T Davis, Sohail Quraishi, Mushtaq Ahmed

**Affiliations:** 1 Orthopaedics, Russell's Hall Hospital, Dudley, GBR

**Keywords:** hip fracture, predictive factors, hip hemiarthroplasty, risk stratified follow-up

## Abstract

Introduction

Up to 19% of patients who undergo surgery for an acute hip fracture are readmitted to the hospital within three months of the index operation. We aimed to identify risk factors for unplanned clinic attendance, readmission, and mortality within the first 12 months postoperatively and subsequently determine if there is a role for routine follow-up.

Method

Patients greater than 65 years old who underwent hip hemiarthroplasty using an uncemented Thompson implant for treatment of a traumatic non-pathological hip fracture were identified from a prospectively maintained database at a single institution between August 2007 and February 2011. Patient demographics, comorbidities, place of residence, mobility status, unplanned attendance to an orthopaedic clinic with symptoms relating to the respective limb, readmission, and mortality were recorded. Regression analysis was performed using the IBM Statistical Package for Social Sciences (SPSS), version 24 (IBM SPSS Statistics, Armonk, NY) with P < 0.05 considered significant.

Results

Five hundred and fifty-four consecutive patients were identified. Unplanned clinic attendance was correlated to age (p = 0.000, B = -0.0159, 95% confidence interval (CI): -0.200 to -0.65), with patients between the ages of 65 - 70 years most likely to require unplanned clinic review postoperatively. The American Society of Anesthesiologists (ASA) grade (p = 0.019, 95% CI: 0.014 to 0.163) and frequency of unplanned outpatient attendance (p = 0.000, 95% CI: 0.120 to 0.284) were significantly associated with increased readmission within 12 months of the index procedure with patients who were regarded as ASA > 2 most likely to require readmission within the first postoperative year.

Conclusion

To our knowledge, this is the first piece of research that identifies causative factors for unplanned clinic attendance and acute readmission during the first postoperative year in acute hip fracture patients treated by hemiarthroplasty. Routine scheduled follow-up of patients based on risk stratification may be effective in reducing the financial burden of unplanned clinic attendance.

## Introduction

Hip fractures are the commonest fracture type experienced by elderly patients and arguably the most debilitating both for the patient and the National Health Service (NHS) [1]. There are currently 80,000 hip fractures admitted annually to United Kingdom (UK) hospitals, costing the NHS an estimated one billion pounds per annum, which equates to 1% of the NHS budget [2]. A recent National Hip Fracture Database (NHFD) report suggests that neck of femur fractures presently account for approximately 20% of occupied hospital beds and the average length of stay for hip fracture patients is 21 days [1].

Morbidity and mortality following a hip fracture is significant, with some studies reporting mortality rates as high as 50% within the first postoperative year [3]. Unfortunately, the incidence of the intracapsular neck of femur (NOF) fractures is increasing in the UK and is projected to continue to rise in the future [4].

According to the NHFD, 49.1% of admitted hip fractures in the UK are displaced intracapsular fractures, and hip hemiarthroplasty is the treatment of choice in 90.5% of these cases [2]. National guidelines on the management of displaced intracapsular fractures recommend using at least an Orthopaedic Data Evaluation Panel-rated (ODEP 3B) cemented implant instead of an unrated Thompson’s implant [5-6]. The Thompson’s prosthesis, however, was designed as a press-fit hemiarthroplasty implant to be used without any cement [7-8].

The vast majority of hip fracture patients are elderly, frail, and comorbid. Coordinated multidisciplinary management of hip fracture cases is advocated by the British Orthopaedic Association and the Royal College of Physicians and is a cornerstone of both the Best Practice Tariff Scheme and the National Institute for Clinical Excellence (NICE) guidelines for hip fracture management [9]. This approach aims to reduce morbidity and mortality by ensuring that hip fracture patients receive optimal surgical, medical, and physiotherapy care throughout the perioperative period. Although overall national 30-day mortality of hip fracture patients has decreased from 10.9% (2007) to 8.5% (2011) since the introduction of the Best Practice Tariff scheme, data from the NHFD demonstrates that treatment of this patient group is still suboptimal [2]. Alarmingly, “the NHFD report found many instances where the hip fracture programme teams are unable to determine what has happened to their patients after they leave the acute unit.” With the incidence and national financial burden of hip fracture management anticipated to rise as adult life expectancy in the UK increases, early multidisciplinary assessment of this patient group and appropriate community follow-up is fundamental to ensuring that postoperative morbidity is reduced, thereby preventing additional costs to the NHS by way of readmissions and clinic re-attendances.

An estimated 19.0% of hip fracture patients who undergo surgery require readmission to the hospital within three months of operation [5]. A recent study by Marmarelis et al. also demonstrated that 4.8% of patients who underwent hip hemiarthroplasty required revision surgery within the first 30 days of the index procedure [10]. Despite this, research to identify patient factors that may be associated with an increased risk of postoperative readmission either for surgical or medical reasons and unplanned clinic attendance is lacking. Readmission and unplanned clinic attendance may indicate negative consequences for the patient but also creates an additional financial burden for the healthcare system.

This study aimed to determine if stratification based on preoperative and 12-month postoperative factors can aid in identifying patients at risk of requiring unplanned clinic review or readmission within 12 months of undergoing hip hemiarthroplasty surgery for an intracapsular neck of femur fracture and if specific preoperative factors increase mortality risk in hip fracture patients treated with hemiarthroplasty. The effect of unplanned clinic attendance, readmission, and reoperation within the first year postoperatively on one-year survival was also assessed.

## Materials and methods

Methods and study design for this patient population have been described previously [11]. A database of patients greater than 65 years who underwent hip hemiarthroplasty using an uncemented Thompson prosthesis for treatment of a traumatic non-pathological hip fracture was prospectively maintained from August 2007 to February 2011. Demographics, preoperative and postoperative data were collected for each case. The number and nature of unplanned attendances to an orthopaedic clinic, readmission, and mortality were recorded during the 12 months following surgery and retrospectively analysed.

Inclusion and exclusion criteria 

Patients over the age of 65 years who underwent hip hemiarthroplasty using an uncemented Thompson prosthesis for treatment of an acute, non-pathological hip fracture were identified for analysis.

Statistical analysis

The IBM Statistical Package for Social Sciences (SPSS), version 24 (IBM SPSS Statistics, Armonk, NY) was used for statistical analysis of the cohort. Regression analysis was performed to identify pre- and postoperative factors associated with unplanned attendance to the clinic, acute readmission to hospital, and survival independently. The level of significance was established at P < 0.05. The Kaplan Meier survival analysis was used to determine the mortality of patients within one year of the index procedure based on age, gender, American Society of Anesthesiologists (ASA) grade, frequency of unplanned attendance to an outpatient clinic, and readmission and reoperation within the first postoperative year.

## Results

Cohort demographics 

One thousand two hundred and eighty-one hip fracture patients were admitted to Russell’s Hall Hospital between August 2007 and February 2011. The study cohort consisted of 554 patients (152 males and 400 females) with a mean age on admission of 83 years (range: 65 - 101 years). The baseline demographics of the cohort are shown in Table 1. 

**Table 1:** Study Cohort Demographics ASA: American Society of Anesthesiologists; LTC: long-term complications

Patient Demographics	Study Cohort (N = 554)
Age Category (%)	
65 - 69 years	17 (3.1%)
70 - 79 years	151 (27.3%)
≥ 80 years	386 (69.7%)
Gender (%)	
Male	154 (27.8%)
Female	400 (72.2%)
ASA Grade (%)	
I	4 (0.7%)
II	88 (15.9%)
III	293 (52.9%)
IV	99 (17.9%)
V	70 (12.6%)
Residence on Admission (%)	
Own home/Sheltered accommodation	397 (71.7%)
Nursing home/Residential care/LTC hospital	132 (23.8%)
Rehabilitation facility	7 (1.3%)
Hospital	15 (2.7%)
Unknown	3 (0.5%)
Walking Ability Indoors on Admission (%)	
Regularly walks without aid	230 (41.5%)
Regularly walks with 1 aid	147 (26.5%)
Regularly walks with 2 aids or a frame	130 (23.5%)
Wheelchair or bedbound	15 (2.7%)
Unknown	32 (5.8%)
Walking Ability Outdoors on Admission (%)	
Regularly walks without aid	121 (21.8%)
Regularly walks with 1 aid	99 (17.9%)
Regularly walks with 2 aids or a frame	17 (3.1%)
Electric buggy	4 (0.7%)
Wheelchair	24 (4.3%)
Never goes outside	59 (10.6%)
Unknown	230 (41.5%)

Unplanned postoperative attendance to clinic 

Seventy-eight patients (14.1%) attended the outpatient clinic on at least one occasion within the first postoperative year. The number and nature of unplanned attendances to the clinic are displayed in Tables 2-3, respectively. The commonest cause for clinic attendance was pain with 53 attendances being attributed to this reason. Patients who had sustained multiple injuries on index admission, who had undergone conversion to total hip replacement following the initial procedure, or who had developed an infection/cellulitis or weakness postoperatively also frequently reattended the clinic for unplanned review. Other reasons for unplanned clinic attendance (46%) included difficulties with gait or orthopaedic issues affecting joints other than the site of operation.

Logistic regression revealed a significant correlation between age and unplanned attendance to an orthopaedic clinic in the first postoperative year with patients aged 65 - 69 years most likely to re-present unexpectedly (p = 0.000, 95% CI: -0.200 to -0.65). Forty-seven percent of patients in this age group attended for at least one unplanned clinic review during the first postoperative year as compared to 16.6% of patients aged 70 - 79 years and only 11.6% of patients > 80 years of age.

**Table 2:** Frequency of Unplanned Attendances to Clinic

Number of Unplanned Attendances to Clinic	Frequency	Percentage
1	45	8.10%
2	17	3.10%
3	8	1.40%
4	4	0.70%
5	4	0.70%

**Table 3:** Reasons for Unplanned Clinic attendance THR: total hip replacement

Reason for Unplanned Attendance	Frequency
Pain	53
Multiple Injuries	9
Postoperative THR Revision	5
Infection/Cellulitis	6
Weakness	2
Other	64

Acute readmission to hospital

Readmission to the hospital within the first postoperative year following hip hemiarthroplasty was significantly correlated with ASA grade and frequency of unplanned attendance to the outpatient clinic. Patients who were considered to be ASA > 2 at the time of the index procedure were more likely to require acute readmission to the hospital (p = 0.019, 95% CI: 0.014 to 0.163) within the first postoperative year. Indeed, 8.4% of patients in our study group who were ASA > 2 required acute readmission to the hospital within 12 months of the index procedure as compared to only 4.3% of patients who were classified as ASA ≤ 2. Increased frequency of attendance to the outpatient clinic was also associated with acute readmission. The patients (17.7%) who attended the clinic on one occasion required future admission to the hospital and 68.7% of patients who required outpatient review on more than one occasion proceeded to require acute admission to the hospital. The reasons for acute admission to the hospital within the first 12-months following the index procedure are outlined in Table 4.

**Table 4:** Reasons for Readmission THR: total hip replacement

Reason for Readmission	Frequency
Dislocation Requiring Closed Reduction	10 (1.8%)
Conversion to THR	24 (4.3%)
Other	9 (1.6%)

Mortality

**Figure 1:**
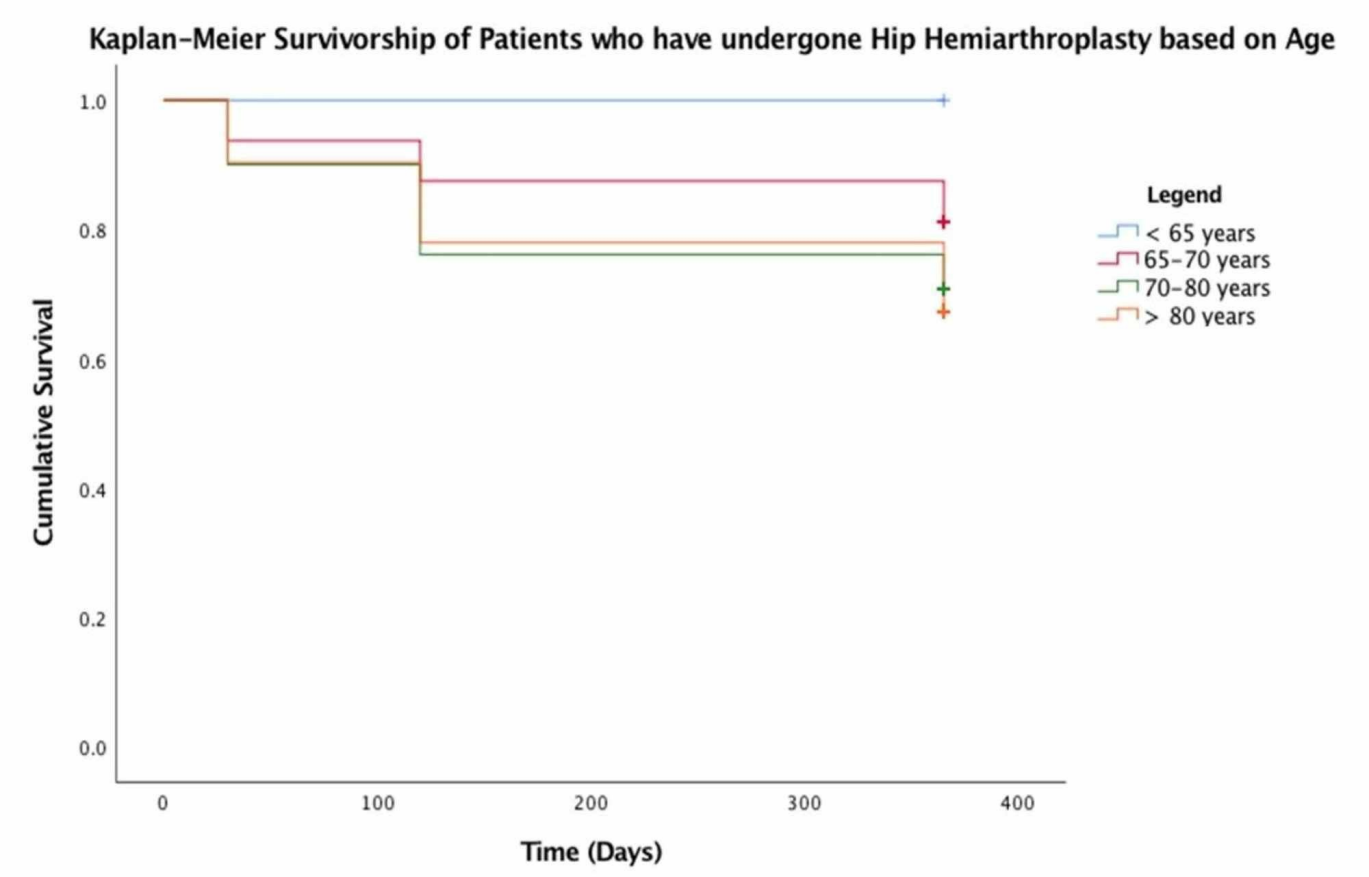
Kaplan-Meier survivorship of patients who have undergone hip hemiarthroplasty based on age

**Figure 2:**
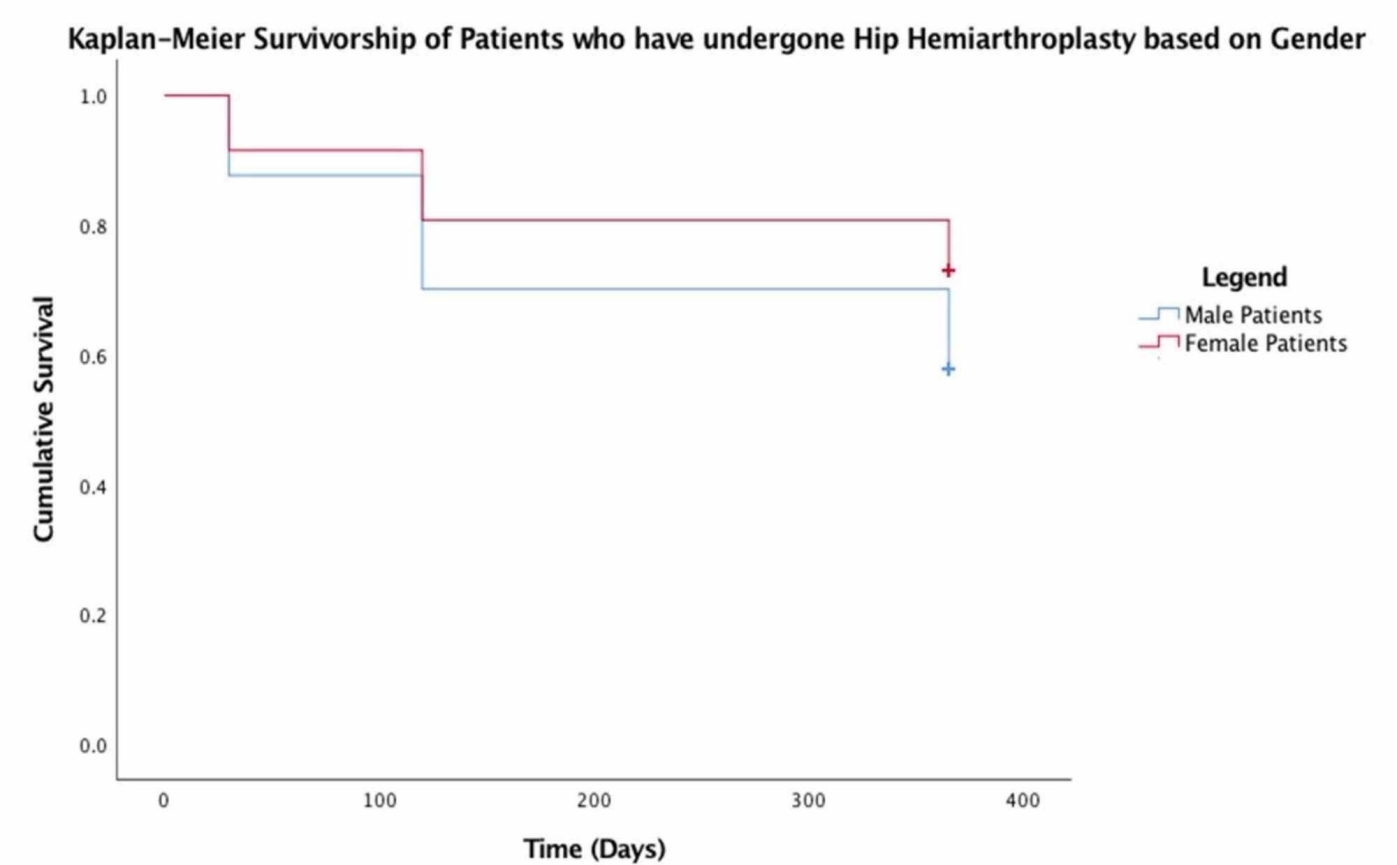
Kaplan-Meier survivorship of patients who have undergone hip hemiarthroplasty based on gender

**Figure 3:**
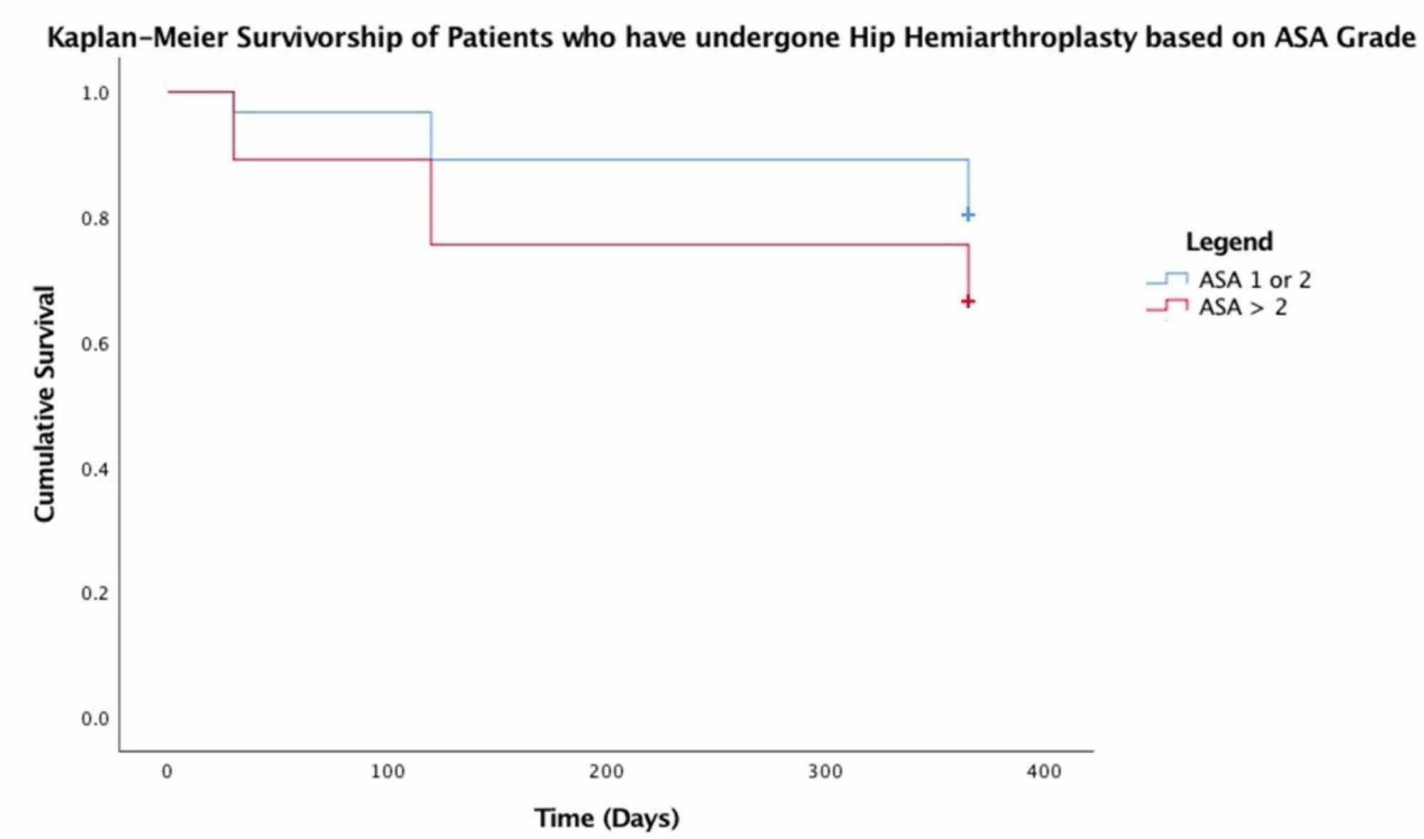
Kaplan-Meier survivorship of patients who have undergone hip hemiarthroplasty based on ASA grade ASA: American Society of Anesthesiologists

No significant predictors were found for mortality following hip hemiarthroplasty for traumatic non-pathological hip fractures in our patient population. However, increased age, an ASA > 2, and male gender all decreased overall survivorship as demonstrated in Figures 1-3, respectively.

**Figure 4:**
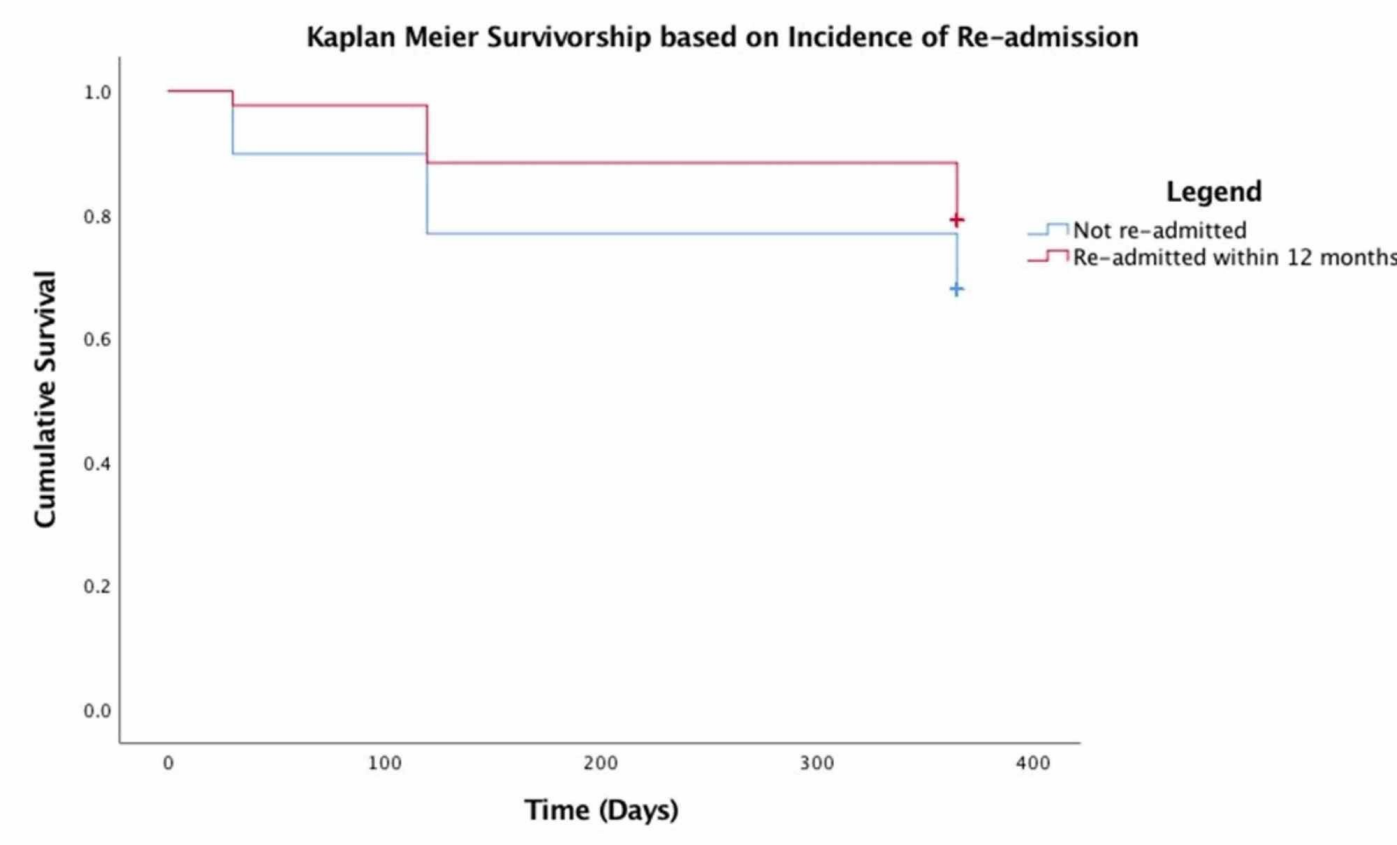
Kaplan-Meier survivorship based on incidence of readmission

**Figure 5:**
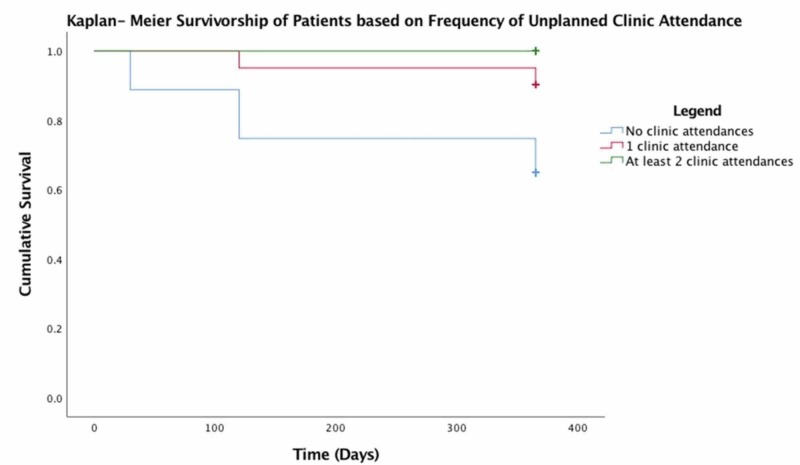
Kaplan-Meier survivorship of patients based on the frequency of unplanned clinic attendance

Interestingly, readmission within one year of surgery increased postoperative survival within the first 12 months. Of the patients who were readmitted to the hospital following the index procedure, 79.1% were alive at 12 months compared to 67.9% of patients who did not require hospital readmission (Figure 4). Patients who attended the clinic on two or more occasions were also more likely to survive the first postoperative year. Only 64.9% of patients who were not reviewed postoperatively in the orthopaedic clinic were alive at one year compared to 90% of patients who attended the clinic on one occasion and 100% of patients who were reviewed more than twice within the first postoperative year (Figure 5). However, there was no difference in mortality between the patients who underwent reoperation following index procedures and the remainder of the patients in the first year postoperatively with 71.6% of patients who did not require reoperation and 71.4% of individuals who did require reoperation, surviving at least 12 months following the index procedure.

## Discussion

Risk factors for unplanned clinic attendance, readmission, and reoperation

Age was shown to be a significant risk factor for unplanned clinic attendance in our patient cohort. ASA grade and frequency of unscheduled clinic attendance postoperatively were both predictors of readmission within the first 12 months. The significance of ASA grade (notably ASA grade > 3) as a predictor for mortality and the need for reintervention has been well established [12-13]. A recent retrospective study demonstrated that patients over 85 years of age, delay of surgery of more than 24 hours, Charlson Comorbidity Index score > 4, the presence of arrhythmia, and preexisting dementia were all independent factors for readmission [14]. Further studies have also suggested that the escalation of a patient’s care from ward-based care to level 2 care postoperatively is associated with a significantly ‘higher ASA risk score’ than patients who only require ward-based care [15]. The ASA grade could conceivably be integrated into a stratification system to aid in preemptively identifying patients that are at greater risk of morbidity and mortality and who are likely to require readmission to the hospital postoperatively.

Predictors of mortality

In our study cohort, Kaplan-Meier survivorship statistics clearly demonstrate that increased age, higher ASA grade, and male gender had a negative effect on survival. Paradoxically, unplanned clinic attendance and hospital readmission appeared to improve overall 12-month survival. Previous studies have demonstrated that readmission confers a significantly increased risk of mortality in this patient group [14]. The reason for this discrepancy is unclear. Further analysis of the readmitted group of patients, which excludes patients who have been readmitted for conversion to THR would perhaps reveal a mortality rate in keeping with the contemporary literature.

There is a paucity of research that directly examines the benefits received by patients who attend an unplanned clinic postoperatively. Although the extent to which clinic review may improve postoperative outcomes is unclear, a recent meta-analysis of the effectiveness of hip fracture schemes concluded that programmes which integrate individual community teams have a profound effect on the overall outcomes of hip fracture patients and have been shown to reduce mortality rates, complications, and readmissions in this patient group; service integration has also been financially beneficial in some cases [16-17]. Indeed, 90% of our study patients who attended at least one unplanned clinic review and 100% of patients who were reviewed on more than one occasion survived their first postoperative year. Further research is required in order to clarify the reasons underpinning the association between clinic review and improved survivorship. Patients who elect to present to an unscheduled clinic may be seeking treatment at an earlier stage than those who do not attend for review, which could effectively improve their first-year survival.

In addition to providing an opportunity for orthopaedic review, planned postoperative clinics for at-risk patients may also be valuable in fostering an environment where further integration of multidisciplinary services can occur and where patients can receive continuous specialist input from orthogeriatric medicine and physiotherapy teams.

Current trends in community follow-up and care

Patients who have previously experienced a hip fracture are known to be at increased risk of subsequent falls [18]. The combination of falls risk and inadequate provision of postoperative community care increases the risk of readmission and mortality in this patient group exponentially. The current NICE guideline encourages “clinical and services governance responsibility for all stages of the pathway of care and rehabilitation, including those delivered in the community” [19]. The 2016 National Hip Fracture Report conceded that establishing the frequency, intensity, form of nursing, and therapist input is difficult and poorly recorded [1]. Therefore, it is likely that the community care received by patients on discharge is currently variable and difficult to quantify.

Recent attempts to ensure the continuity of care of hip fracture patients following discharge from hospital include the Physiotherapy Hip Fracture Sprint Audit (PHFSA) produced in collaboration with the NHFD [20]. There were three key recommendations detailed in the PHFSA report. First, the document highlighted the merits of early mobilisation and suggests that this is best achieved through a collaborative multidisciplinary team working to ensure early progress in rehabilitation. Second, the report advocated intensive rehabilitation to maximise the number of patients discharged directly home and increased focus on modalities such as strength, balance, and stamina, in addition to mobility. Finally, the audit report emphasised the importance of local governance and continuous quality improvement, including conducting monthly hip fracture meetings, submitting regular Hip Sprint data, and identifying areas within the physiotherapy services and allied staffing which may affect rehabilitation such as service provisions. Notably, the Hip Sprint Audit also identified two major findings related to current community care. Alarmingly, only 21% of patients started a home rehabilitation programme within one week of discharge with 10% of community physiotherapists not receiving any handover from their hospital counterparts. In addition, only 20% of services provided physiotherapy on more than four days of the patient’s first week at home with an average waiting time of 15 days to start therapy services at home. The PHFSA identified a number of areas for improvement within community care. Optimising community care and physiotherapy would certainly reduce unplanned clinic attendance and may reduce hospital admission in certain patient populations.

The economic impact of unplanned clinic attendance

In our study population, approximately 14.1% of individuals who underwent hip hemiarthroplasty required at least one unplanned clinic attendance postoperatively. 

With the estimated cost of a consultant-led outpatient appointment being approximately 151 pounds [6], we can estimate the total cost for our cohort from unplanned clinic attendance alone to be £20,989 pounds. Careful selection of those benefitting from planned outpatient follow-up may significantly reduce the burden of the unplanned clinic and hospital readmission costs.

## Conclusions

We have identified a series of risk factors, including age and ASA grade, which could be predictive of unplanned clinic attendance during the first postoperative year in patients treated by uncemented hip hemiarthroplasty following an acute hip fracture. Routine, scheduled follow-up of patients based on risk stratification may be effective in reducing the financial burden of unplanned clinic attendance, hospital readmission, and reoperation. It may additionally improve the integration of services and continuity of care received by hip fracture patients following discharge in the community. We propose further research on this to fully evaluate the potential survival benefits associated with clinic review and readmission demonstrated in our patient cohort.
